# Lung nuclear protein in testis carcinoma in an elderly Korean woman: A case report with cytohistological analysis

**DOI:** 10.1111/1759-7714.13438

**Published:** 2020-04-22

**Authors:** Hwa Jin Cho, Hyun‐Kyung Lee

**Affiliations:** ^1^ Department of Pathology Inje University Busan Paik Hospital Busan, Republic of Korea; ^2^ Division of Pulmonary and Critical Care Medicine, Department of Internal medicine Inje University Busan Paik Hospital Busan, Republic of Korea

**Keywords:** Case report, immunohistochemical analysis, NUT carcinoma, pulmonary

## Abstract

Nuclear protein in testis (NUT) carcinoma is a rare, aggressive carcinoma that is a diagnostic challenge for pathologists. Here, we report a case of NUT carcinoma in a 63‐year‐old woman with uncommon immunohistochemical results. The initial bronchoscopic biopsy revealed a poorly differentiated carcinoma with p63 immunohistochemical stain positivity. However, the cytomorphological features of the pleural fluid were unusual. Immunohistochemical staining of the pleural fluid revealed diffuse positivity for vimentin and focal positivity for cytokeratin and neuroendocrine markers. Because of chemoresistance, other malignancies, including sarcomatoid carcinoma, combined small cell carcinoma, and an unusual form of NUT carcinoma, were considered as differential diagnoses. The diagnosis of NUT carcinoma was confirmed using NUT‐specific antibodies and fluorescence in situ hybridization. The current case was a diagnostic challenge because of the poorly differentiated cytomorphology and uncommon immunohistochemical results. Pathologists and clinicians should consider NUT carcinoma in the differential diagnosis, as this malignancy has a dismal prognosis and needs to be diagnosed accurately for the most effective treatment.

**Key points:**

- Metastatic NUT carcinoma can show diffuse vimentin positivity and focal neuroendocrine marker positivity. NUT carcinoma can be misdiagnosed as basaloid squamous cell carcinoma in routine diagnosis, especially in older‐aged patients.
- This study was a diagnostic challenge because of the poorly differentiated cytomorphology and uncommon immunohistochemical results for NUT carcinoma. Pathologists should differentially diagnose NUT carcinoma when rare cytohistological features are observed at any age.

## Introduction

Nuclear protein in testis (NUT) carcinoma is a rare, aggressive carcinoma involving NUT rearrangement. It may arise at any age (0.1–81.7 years), but on the basis of the median patient age, it usually occurs in childhood or young adulthood (ie, 16–24 years).^1^, ^2^, ^3^, ^4^ NUT carcinoma is refractory to conventional chemotherapy and has a dismal prognosis.^2^, ^3^ NUT carcinoma is often misdiagnosed because of its various morphologies, leading to inappropriate treatment. Here, we report a case of NUT carcinoma of the lung in an elderly woman with uncommon immunohistochemical results.

## Case report

**Figure 1:**
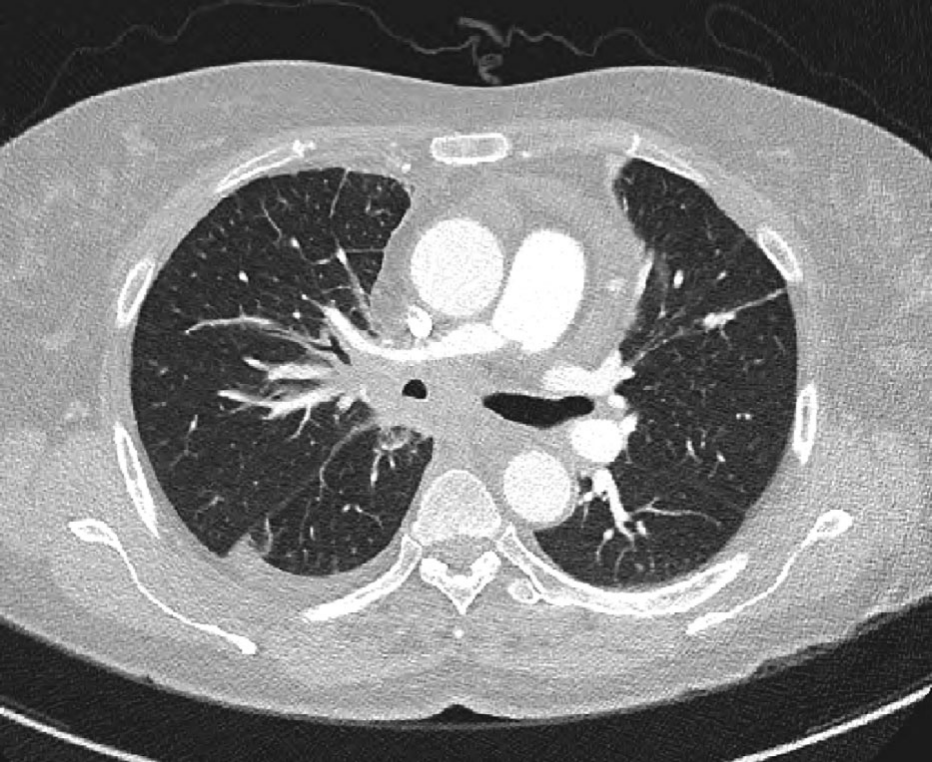
Small residual amount of right pleural effusion with subsegmental atelectasis in the right lower and middle lobe. Bronchial wall thickening and stenosis with peribronchial soft‐tissue infiltration along the right main and intermediate as well as all three lobar bronchi. Ill‐defined soft tissue infiltrations at the subcarinal and lower paratracheal area, suggestive of endobronchial central lung cancer with obstructive pneumonitis.

A 63‐year‐old woman presented with complaints of right flank pain, cough, and breathing difficulties for two months. Her initial laboratory test results were normal. Initial computed tomography (CT) scan showed a large amount of right pleural effusion and consolidation in the right middle lobe. On the second chest CT after percutaneous drainage, suspicious findings indicative of endobronchial central lung cancer with obstructive pneumonitis were observed (Fig 1).

**Figure 2:**
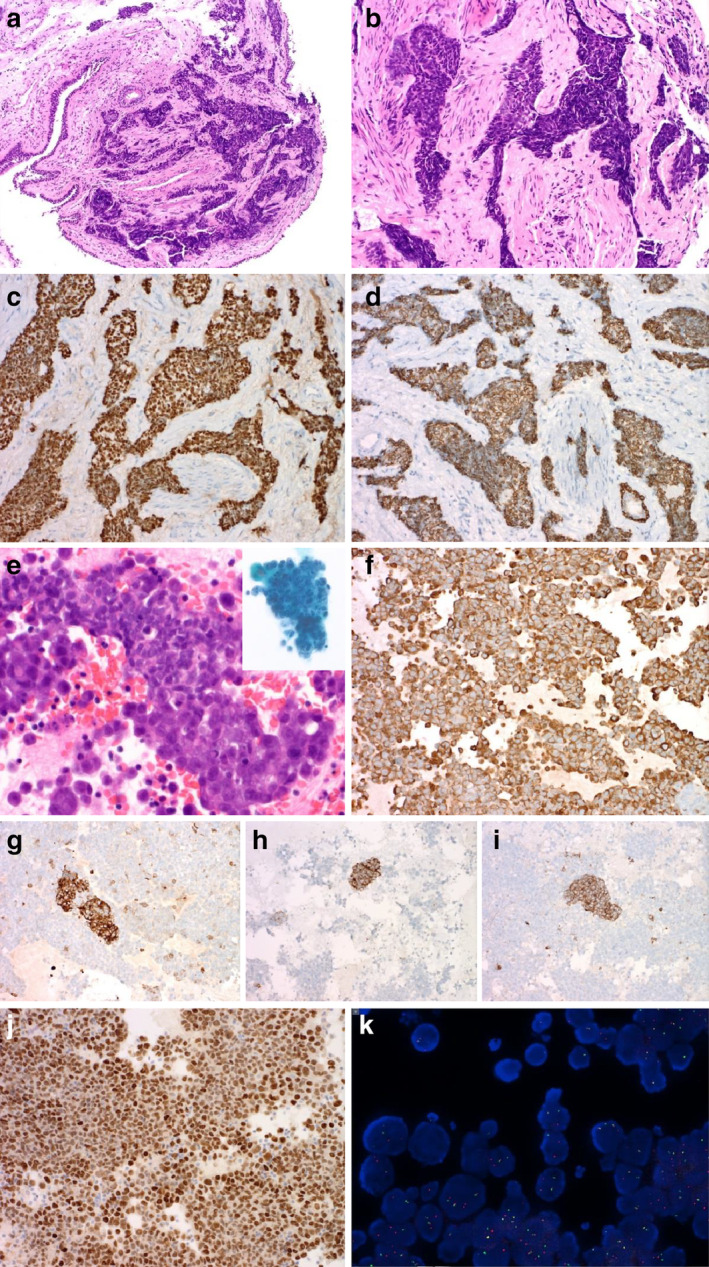
Pathological findings of NUT carcinoma. **a**–**d**: Microscopic findings of tumor obtained from bronchoscopic biopsy. (**a**) Nests of small‐to‐intermediate‐sized monomorphic cells infiltrating the bronchial mucosa. (**b**) Frequent nuclear molding and prominent nucleoli. (**c**) Diffuse strong nuclear p63 positivity. (**d**) Diffuse positivity of CK5/6. **e**–**i**: Pleural fluid cytomorphology observed on immunohistochemical staining. (**e**) Hyperchromatic malignant cell clusters in the liquid‐based preparation (inlet) and cell block. (**f**) Diffuse vimentin positivity. Focal positivity for (**g**) pancytokeratin, (**h**) chromogranin, and (**i**) synaptophysin. (**j**) and (**k**) NUT evaluation with pleural fluid effusion. (**j**) Immunohistochemical staining of NUT antibody showing diffuse positivity. (**k**) Frequent chromosomal translocation in the *NUT* gene (NUTM1), as observed using the fluorescence in situ hybridization break‐apart probe. (Original magnification and stain: a: ×100; b, c, and d: ×200; e–i: ×200; a and b: H&E; c: p63; d: CK5/6; e: H&E; f: vimentin; g: pancytokeratin; h: chromogranin; i: synaptophysin; j: NUT)

A bronchoscopic biopsy specimen revealed nests of small‐to‐intermediate‐sized, monomorphic cells with vesicular nuclei. Frequent nuclear molding and distinct nucleoli were observed (Fig 2a,b). Results of immunohistochemical staining are shown in Table 1 and Fig 2c,d. Because cells were positive for p63 and CK5/6, an initial diagnosis of basaloid squamous cell carcinoma (SqCC) was made. Positron emission tomography‐CT (PET‐CT) showed no extrathoracic organ metastasis.

**Table 1:** Immunohistochemical analysis results of the primary and metastatic tumors in the pleural fluid

	Positivity	Extent
Initial tumor from the RML bronchus		
TTF‐1	Negative	NA
Napsin A	Negative	NA
P63	Positive	Diffuse
Cytokeratin 5/6	Positive	Diffuse
CD56	Negative	NA
Pancytokeratin	Positive	Diffuse
Residual tumor from the RML bronchus after 11 months		
TTF‐1	Negative	NA
P63	Positive	Focal
CD56	Positive	Focal
Cytokeratin 7	Positive	Diffuse
Metastatic tumor in the pleural fluid after 11 months		
TTF‐1	Negative	NA
P63	Positive	Focal
CD56	Positive	Focal
Chromogranin	Positive	Focal
Synaptophysin	Positive	Focal
Pan‐cytokeratin	Positive	Focal
Vimentin	Positive	Diffuse
Calretinin	Negative	NA
HBME‐1	Negative	NA

NA, not applicable; RML, right middle lobe.

The tumor progressed despite multiple rounds of chemotherapy and showed metastasis to abdominal lymph nodes and the liver; therefore, a second biopsy and percutaneous drainage were performed 11 months later. Liquid‐based preparation and cell‐block analysis of the pleural fluid were performed (Fig 2e–i). The tumor cells showed a singly scattered or tightly clustered pattern. The nuclei were hyperchromatic, and the nuclear chromatin pattern was coarse, with prominent nucleoli. The tumor was diffusely positive for vimentin and focally positive for pancytokeratin, p63, and neuroendocrine markers. A diagnosis of mixed sarcomatoid, squamous, and small cell carcinoma was made. Differential diagnoses including unusual NUT carcinoma and sarcomatoid malignant mesothelioma were considered.

Additional NUT immunohistochemical staining (1:100, C52B1, Cell Signaling Technology, Danvers, MA, USA) revealed positivity in the nuclei (Fig 2j). Chromosomal translocation in the *NUT* gene (NUTM1) was observed using break‐apart fluorescence in situ hybridization (FISH) (Fig 2k). NUT carcinoma was finally diagnosed.

Clinically, the tumor progressed over 13 months, and the last chest CT showed progressive lung cancer with extensive pleural metastasis. The patient was discharged to a local hospital for supportive care, but she was lost to follow‐up.

## Discussion

This case shows unusual immunohistochemical results associated with NUT carcinoma. The patient's older age and the difference in cytohistological features of primary and metastatic carcinoma in the pleural fluid resulted in difficulty in making an accurate diagnosis.

A recent study of NUT carcinoma in Korea reported a median age of 48.0 years (range, 8–73 years), which was higher than that reported in Western studies (30 years).^5^, ^6^ NUT carcinoma is extremely aggressive and has a dismal prognosis. Diagnosing NUT carcinoma has been challenging, primarily because its cytological and histological morphologic features vary and overlap with those of some poorly differentiated or undifferentiated malignancies. The typical histological features are sheets and nests of monomorphic small‐to‐intermediate‐sized round‐oval cells. The amount of cytoplasm is scant‐to‐moderate, and the nuclear‐to‐cytoplasmic ratio is high, with frequent mitoses. Nuclei are vesicular to hyperchromatic. Additional histological findings such as mesenchymal differentiation^7^ have also been reported.

The main differential diagnoses in the current case were small cell carcinoma, basaloid or poorly differentiated SqCC, the small cell variant of SqCC,^8^ combined small cell carcinoma and sarcomatoid carcinoma (in the pleural fluid), and other carcinomas showing a small round cell morphology, including NUT carcinoma.^9^ Immunohistochemical staining^5^, ^6^ and cytological specimens,^8^, ^9^, ^10^ along with radiological diagnostic methods like PET‐CT, are useful for differential diagnosis of NUT carcinoma of the lung. NUT carcinoma is usually positive for cytokeratins and p63 and negative for neuroendocrine markers.^9^ However, spotty AE1/AE3 and CD138 staining and diffuse vimentin positivity have been reported.^11^ A case of parotid gland NUT carcinoma also showed CD56 positivity^7^; that case had a malignant heterologous mesenchymal component, a possible form of epithelial‐mesenchymal transition (EMT). In our case loss of epithelial marker reactivity focally and gain of vimentin positivity diffusely in the pleural fluid were observed, possibly because of the EMT that is known to be associated with tumor metastasis and a poor prognosis in many cancers.^12^, ^13^ Thus, vimentin positivity does not rule out NUT carcinoma.

Although the incidence of NUT carcinoma is increasing, it remains a diagnostic challenge, particularly with cytology specimens. Therefore, further research on the cytopathology of NUT carcinoma is needed. Moreover, testing for specific monoclonal NUT antibodies should be performed in all cases of poorly differentiated carcinomas with p63‐positive cancer across all ages. This case is valuable in that both histological and cytological features including rare immunohistochemical results were delineated in the elderly woman.

## Disclosure

There are no conflicts of interest to declare.
